# Intersecting household-level health and socio-economic vulnerabilities and the COVID-19 crisis: An analysis from the UK^☆^

**DOI:** 10.1016/j.ssmph.2020.100628

**Published:** 2020-07-02

**Authors:** Júlia Mikolai, Katherine Keenan, Hill Kulu

**Affiliations:** University of St Andrews and ESRC Centre for Population Change, Irvine Building, North Street, St. Andrews, KY16 9AL, UK

**Keywords:** Inequalities, Health, COVID-19, Household dynamics, United Kingdom, Principal components analysis

## Abstract

The effects of COVID-19 are likely to be socially stratified. Disease control measures introduced during the COVID-19 pandemic mean that people spend much more time in their immediate households, due to lockdowns, the need to self-isolate, and school and workplace closures. This has elevated the importance of certain household–level characteristics for individuals’ current and future wellbeing. The multi-dimensional poverty and health inequalities literature suggests that poor health and socio-economic conditions cluster in the general population, which may exacerbate societal inequalities over time. This study investigates how COVID-19-related health- and socio-economic vulnerabilities co-occur at the household level, and how they are distributed across household types and geographical areas in the United Kingdom. Using a nationally representative cross-sectional study of UK households and individuals and applying principal components analysis, we derived summary measures representing different dimensions of household vulnerabilities critical during the COVID-19 epidemic: health, employment, housing, financial and digital. Our analysis highlights four key findings. First, although COVID-19-related health risks are concentrated in retirement-age households, a substantial proportion of working-age households also face these risks. Second, different types of households exhibit different vulnerabilities, with working-age households more likely to face financial and housing precarities, and retirement-age households health and digital vulnerabilities. Third, there are area-level differences in the distribution of household-level vulnerabilities across England and the constituent countries of the United Kingdom. Fourth, in many households, different dimensions of vulnerabilities intersect; this is especially prevalent among working-age households. The findings imply that the short- and long-term consequences of the COVID-19 crisis are likely to significantly vary by household type. Policy measures that aim to mitigate the health and socio-economic consequences of the COVID-19 pandemic should consider how vulnerabilities cluster and interact with one another both within individuals and different household types, and how these may exacerbate already existing inequalities.

## Introduction

Emerging evidence on the social epidemiology of COVID-19 suggests that infections and deaths from the disease operate along existing axes of social inequalities, and that individuals from ethnic minorities, poorer socioeconomic backgrounds and deprived areas are more likely to suffer (Jordan & Adab, 2020; Williamson et al., 2020). While individual risk factors have received much attention, it is unclear how such inequalities occur and intersect at the household level and how this might influence the short- and long-term consequences of the pandemic. Physical distancing measures including household lockdowns, self-isolation for high-risk individuals, and school and workplace closures mean that more time is spent in the household, which have led to concerns over financial, physical and psychological effects as well as potentially widening societal and health inequalities (Douglas et al., 2020). Some household characteristics have become elevated in importance for wellbeing, such as access to a garden or safe outdoor space, technology and internet connectivity, and lack of household crowding, especially because under the given circumstances, these factors are less likely to be mitigated by interactions with school, work and community contexts (Armitage & Nellums, 2020; Holmes et al., 2020; Van Lancker & Parolin, 2020; Wang et al., 2020). Furthermore, global economic slowdown and rising unemployment (Karanikolos et al., 2013) may interact with these disparities and exacerbate already existing health- and socio-economic inequalities as the pandemic progresses.

COVID-19 policy advice and research has focussed on mitigating and identifying individual-level health risks, without much consideration of how old and young individuals are nested within different household structures and how their opportunities to follow government guidelines might be limited by household and housing characteristics. For example, in March 2020 the UK government advised ‘extremely vulnerable’ individuals to shield themselves and self-isolate (often along with their entire household) for a period of 12 weeks, but due to different household situations,this may have had radically different implications for how they could protect themselves and organise their lives. Recently, leading public health experts have argued that individual-level vulnerabilities might interact to lead to poorer health outcomes during the COVID-19 crisis (Douglas et al., 2020). We also know from the health inequalities literature that ill health is more common among those suffering other social deprivations, such as poorer housing, overcrowding, financial precarities and social exclusion (Marmot, 2020; Sacker et al., 2017). Therefore, self-isolation may negatively impact other dimensions of disadvantage, not only for the ‘extremely vulnerable’ individuals but also for their household members. A much larger proportion of the population might be considered ‘high risk’ suffering from chronic conditions that make the chances of COVID-19 complications, such as severe respiratory illnesses, more likely. Intersectionality approaches emphasise how combinations of characteristics may worsen possible health and social outcomes (Bauer, 2014). Even in households where no one suffers from immediate COVID-19 health vulnerabilities, intersecting social and economic vulnerabilities might exacerbate or contribute to the development of further vulnerabilities over time (Douglas et al., 2020). Understanding at-risk groups is crucial to be able to prevent a health- and socio-economic crisis in the short- and long-term.

In this study, we investigate household-level health and socio-economic vulnerabilities and how they co-occur across different household types and geographic areas, using cross-sectional data from a nationally representative household survey in the United Kingdom. Our principal aim is to identify intersecting dimensions of household vulnerability, to investigate how they vary by household type and region, and to determine the importance of intersecting vulnerabilities and household structure when mitigating the consequences of the COVID-19 crisis.

## Methods

### Data

We used information from the latest available wave (wave 9) of the UK Household Longitudinal Study (University of Essex Institute for Social and Economic Research, 2019) (UKHLS), from 2017 to 2019, which interviewed approximately 36,000 individuals nested in approximately 20,000 households. We dropped 622 households (3%) due to missing values on the variables used in the analyses. Our analytical sample consists of 19,425 households.^1^

### Variables

#### Indicators of household-level vulnerability

All vulnerability indicators are calculated at the household-level. For some indicators, information is only available at the individual level. In this case, we have calculated measures at the household-level indicating whether at least one person in the household has a given vulnerability.

We used binary indicators to measure *digital and connectivity* features of the household: whether the household reported having a home internet connection, and whether they owned a laptop, PC, netbook, tablet, or other type of computer.

*Housing conditions* were captured through three indicators: whether the household lived in a flat (a proxy for lack of access to outdoor space), whether the accommodation was privately rented, and whether the household lived in overcrowded conditions. Overcrowding was defined as having more than 1 person per room (excluding bathrooms and kitchens) in the dwelling; a measure shown to have equal validity compared with more complex overcrowding metrics (Cable & Sacker, 2019).

*Employment conditions* of the household were captured using four dummy indicators of whether anyone in the household was unemployed, self-employed, worked part-time, or was employed on a temporary contract.

The *financial conditions* of the household were measured using two indicators. First, we used a binary variable indicating whether the household reported being in payment arrears (either being behind on paying bills at the time of the interview or having been behind on housing payments in the last 12 months). Second, we created a dummy indicator for households with relative low income defined as households whose net equivalized household income was lower than 60% of the median net equivalized household income following the definition used by the Department for Work and Pensions (Department for Work and Pensions, 2020).

We defined two *health indicators*. First, we derived an individual-level indicator for health conditions indicating a higher risk of COVID-19 complications. In wave 1 of UKHLS (2009–2011) (or in case of new entrants the first time they were interviewed), respondents report if they have ever been doctor-diagnosed with a list of health conditions^2^. In subsequent waves, respondents were asked about any new health conditions diagnosed since last interview. Using the current NHS guidance^3^ which provides a list of conditions indicating high risk, we created a binary indicator of whether the respondent reported ever being diagnosed with any of the following: asthma, congestive heart failure, coronary heart disease, angina, heart attack or myocardial infarction, stroke, emphysema, chronic bronchitis, liver condition, diabetes, cancer, or hypertension. As these are chronic illnesses, we assumed that if an individual reported a condition in a prior wave, the condition also persists to later waves. The prevalence of these individual conditions at the household level is shown in Appendix Table A1. The most common illnesses were hypertension (27%) and asthma (17%). To capture those individuals who had more severe versions of these conditions, we combined information from this variable and one which indicates whether individuals suffered from any long-standing physical or mental impairment, illness, or disability. The health indicator variable takes the value of 1 if there is an individual in the household who has ever had any of the listed conditions and who also reported to have a long-standing condition in wave 9. The second health indicator takes the value of 1 if there is an individual in the household who reported that their current job status is ‘long-term sick or disabled’.

Aggregating information from individual-level level data for households might mean that larger households are more likely to experience the given vulnerability indicator than those with fewer members. However, in the vast majority of households, only one person experiences the individual-level indicators of vulnerabilities (see Appendix Table A2). For example, only in around 3% of the households are there more than one part-time employed individuals (except in retirement-age households), and just over 1% of the households contains more than one self-employed individuals. When it comes to the health dimension, almost 10% of multi-generational households include more than one person with health issues; this proportion is 6% among retirement-age households. Additional analyses (not shown) stratified by the size and age structure of households have confirmed that these compositional differences within households do not drive the results of the paper.

#### Household type and area type

Based on the age of and relationships between household members, we categorised households into five types: single-parent households with children (15 years and under), working-age (below state pension age^4^) adult households with children, working-age adult households without children, multi-generational households (i.e. at least one working-age adult and one over state pension age, not necessarily related), and retirement-age households, containing only those who are at least of state pension age.

The area type variable divided UK households into the North of England (North East, North West, Yorkshire and Humber, East and West Midlands), South of England (East of England, South East and South West), London, Wales, Scotland, and Northern Ireland.

### Analysis

After describing the distribution of household-level vulnerability indicators and household type, we used principal components analysis (PCA) to establish different dimensions of household vulnerabilities (Abdi & Williams, 2010). PCA has been widely used and validated in the multidimensional poverty literature as a data reduction technique when analysing a set of binary variables (Vyas & Kumaranayake, 2006). The number of principal components was selected such that the eigenvalue was larger than 1 for each principal component (Matsunaga, 2010). After applying promax oblique rotation,^5^ we determined which indicator belongs to which principal component by applying the commonly agreed criteria that the factor loading needs to be above 0.32 (Comrey & Lee, 1992; Tabachnick & Fidell, 2007; Zwick & Velicer, 1982). Then, to analyse how different dimensions of vulnerabilities are distributed across household types and different geographical areas, we calculated dummy variables for the top 25% of the scores on each principal component to indicate households who are most vulnerable on each dimension. All estimates were weighted using cross-sectional household weights.

## Results

**Fig. 1:**
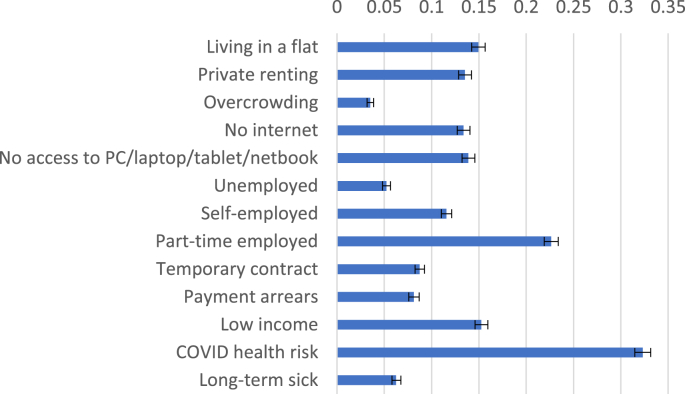
Proportion of households who experience different types of vulnerabilities. *Source*: Authors' calculations using data from the UK Household Longitudinal Study, wave 9 (2017/2019). Weighted estimates. Whiskers indicate 95% confidence intervals.

First, we describe the indicators used in the analyses. Fig. 1 shows the proportion of households who experience different types of vulnerabilities. The most striking finding is that 32% of households contain at least one individual with a health condition that puts them under a higher risk of experiencing severe health consequences of COVID-19 if infected. This was more common in retirement-age and multi-generational households (both 49%) but ranged between 16% and 26% in working-age households (Appendix Table A3). It was also notable that single-parent households were the most financially precarious: approximately one third experienced payment arrears and low income (Appendix Table A3). Second, around 23% of households have at least one individual who is a part-time employee. These households might be at higher risk of COVID-19-induced financial vulnerabilities as they might have fewer savings than households with only full-time employed individuals. Furthermore, between 10% and 15% of households live in a flat, live in privately rented dwellings, contain at least one self-employed individual, have no access to the internet, and have no access to a computer, laptop, tablet or netbook in the household. A smaller share of households experience overcrowding (3.5%), unemployment (5%) or temporary employment (9%), have payment arrears (8%) or contains a person who cannot work due to long-term health conditions or disabilities (6%).

**Fig. 2:**
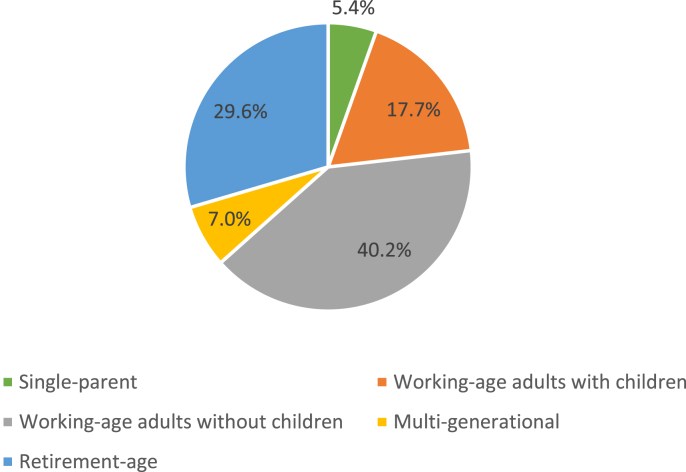
Proportion of different household types in the UK. *Source*: Authors' calculations using data from the UK Household Longitudinal Study, wave 9 (2017/2019). Weighted estimates.

Next, we show the distribution of different types of households (Fig. 2). The majority of households in the UK (40%) are working-age adult households without resident children; the next largest (30%) type of households are retirement-age households. These are followed by working-age adult households with resident children (18%), multi-generational households (7%), and single-parent households (5%). In terms of household size, almost 60% of retirement-age households contained one person and living alone was also common among working-age households without children (42%) (results not shown, but available on request). Table A3 and A4 in the Appendix show how these vulnerability indicators are distributed across different household types and geographic areas, respectively.

To reduce the number of vulnerability indicators, we used principal components analysis (PCA). The results revealed five distinct dimensions of household vulnerabilities (Table 1): digital (access to internet, access to computer), financial (overcrowding, unemployment, low household income, payment arrears), employment (self-employed, part-time, and temporary employment), housing (living in flat and private renting), and health (COVID-19 health risk and long-term illness). The factor scores show the correlations between each vulnerability indicator and each principal component. For example, access to the internet and access to a computer are equally highly correlated with the digital vulnerabilities component, whereas having someone unemployed in the household is more highly correlated with the financial vulnerabilities component than payment arrears. Table A3 in the Appendix also shows how common each vulnerability indicator is across different household types, which reveals how some individual indicators drive the results for some types of households. For example, among single-parent households 6% experience overcrowding, 11% are unemployed, and 30% have low income. Thus, for the financial dimension, having low income is the most important indicator for single-parent households.

**Table 1:** Rotated factor scores from Principal Components Analysis.

	Digital	Employment	Financial	Housing	Health
Living in a flat	0.111	−0.029	−0.081	**0.630**	0.041
Private renting	−0.042	−0.020	−0.085	**0.657**	−0.134
Overcrowding	−0.086	0.061	**0.403**	0.118	−0.008
Unemployed	−0.050	−0.078	**0.626**	−0.152	−0.017
Self-employed	0.014	**0.522**	0.003	−0.065	−0.071
Part-time employed	−0.034	**0.561**	0.011	−0.050	−0.037
Temporary contract	0.036	**0.631**	−0.082	0.034	0.062
No internet	**0.688**	0.010	0.016	0.021	−0.002
No access to PC/laptop/tablet/netbook	**0.685**	0.012	0.027	0.034	0.003
Low income	0.163	−0.027	**0.577**	−0.067	−0.086
Payment arrears	−0.041	0.076	**0.324**	0.306	0.200
COVID-19 health risk	0.063	0.001	−0.033	−0.197	**0.660**
Long-term sick	−0.048	−0.007	−0.032	0.070	**0.703**

*Source*: Authors' calculations using data from the UK Household Longitudinal Study, wave 9 (2017/2019).

*Note*: Boldface indicates factor loadings over 0.32.

**Fig. 3:**
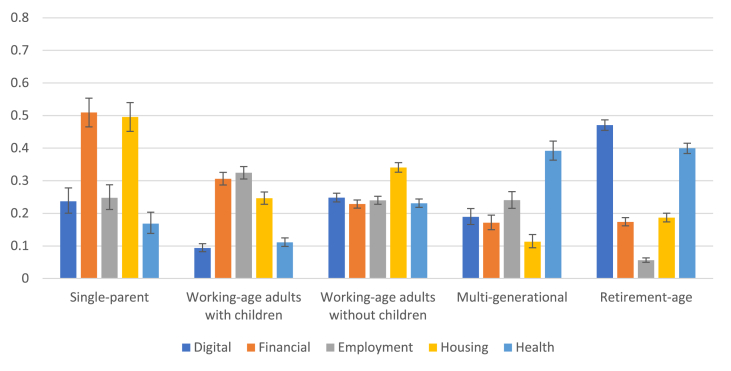
Proportion of households who experience the most severe vulnerabilities (top 25% of each principal component score) in different dimensions by household type. *Source*: Authors' calculations using data from the UK Household Longitudinal Study, wave 9 (2017/2019). Weighted estimates. Whiskers indicate 95% confidence intervals.

**Fig. 4:**
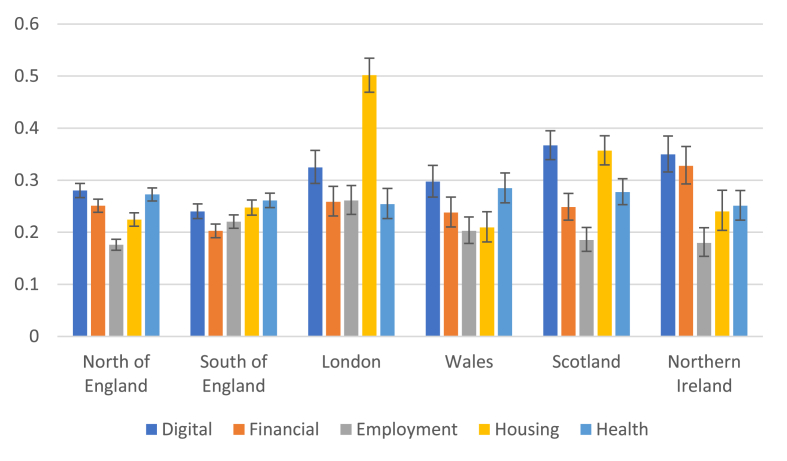
Proportion of households who experience the most severe (top 25% of each principal component score) vulnerabilities in different dimensions by geographical area. *Source*: Authors' calculations using data from the UK Household Longitudinal Study, wave 9 (2017/2019). Weighted estimates. Whiskers indicate 95% confidence intervals.

In the following parts of the analysis, we use these five dimensions of vulnerabilities and analyse how being in the top quartile (most severe vulnerabilities) for these household-level vulnerabilities are distributed across different household types (Fig. 3) and geographical areas (Fig. 4) of the United Kingdom.

*Single-parent households* are most likely to experience the most severe financial and housing vulnerabilities (51% and 50% of single-parent households, respectively). Additionally, 24% of these households face severe digital vulnerabilities. *Working-age adult households with children* are most likely to experience severe vulnerabilities on the employment (32%), financial (31%), and housing (25%) dimensions. Around 10% of these households face severe digital and health vulnerabilities. *Working-age adult households without children* are most likely to experience housing precarities (34%) (compared with other precarities) and are approximately equally vulnerable on all other dimensions (around 22–25%). The most commonly experienced vulnerability in *multi-generational households* is health vulnerabilities (39%), and a large share of these households also experience severe employment-related disadvantage (24%). Around 19% of these households experience severe digital vulnerabilities, 17% faces financial vulnerabilities and 11% severe housing issues. Additional analysis (not shown but available upon request) revealed that the composition of multi-generational households might also matter for health, employment, and housing vulnerabilities. A somewhat higher proportion of multi-generational households experience health vulnerabilities where several pensioners are present compared to households with just one pensioner. Additionally, employment vulnerability is somewhat more prevalent in households with more working-age individuals. Last, severe housing vulnerabilities are less likely in households headed by pension-age individuals (pension-age individuals living with children only or with one working-age adult). Approximately 40% of *retirement-age households* experience severe health vulnerabilities and 47% digital vulnerabilities, but a large share of them also face severe financial (17%) and housing (19%) precarities.

We also find some differences in the types of vulnerabilities households experience by geography (Fig. 4). Comparing the North and South of England, households in the North experience higher levels of severe digital and financial vulnerabilities whereas those in the South are somewhat more likely to experience severe employment vulnerabilities. London stands out; households in London are particularly exposed to severe housing and digital vulnerabilities when compared to other areas of England. Households in Wales are very similar to those in England although they experience somewhat higher levels of digital and health vulnerabilities. Households in Scotland are most exposed to severe digital and housing vulnerabilities whereas in Northern Ireland, digital and financial vulnerabilities are the most prevalent.

Many households experience intersecting vulnerabilities (Table 2); this is especially prevalent among working-age households. Among single-parent households, a large proportion (>30%) of households who face severe health, digital, and employment precarities, also face financial and housing disadvantage and many (62%) who experience severe financial disadvantage also experience housing precarity. Among working-age households (with and without children), severe health and digital vulnerabilities intersect with employment, financial, and housing disadvantage. At the same time, financial precarities intersect with housing vulnerabilities. Among working-age households without children, severe health vulnerabilities intersect with severe digital, financial, and housing disadvantage. Intersecting vulnerabilities are less prevalent among multi-generational and retirement-age households. In both, severe digital vulnerabilities overlap with severe financial disadvantage. In addition, 44% of retirement-age households who have severe health vulnerabilities also have digital vulnerabilities and 33% of those who experience digital vulnerabilities also face housing disadvantage.

**Table 2:**
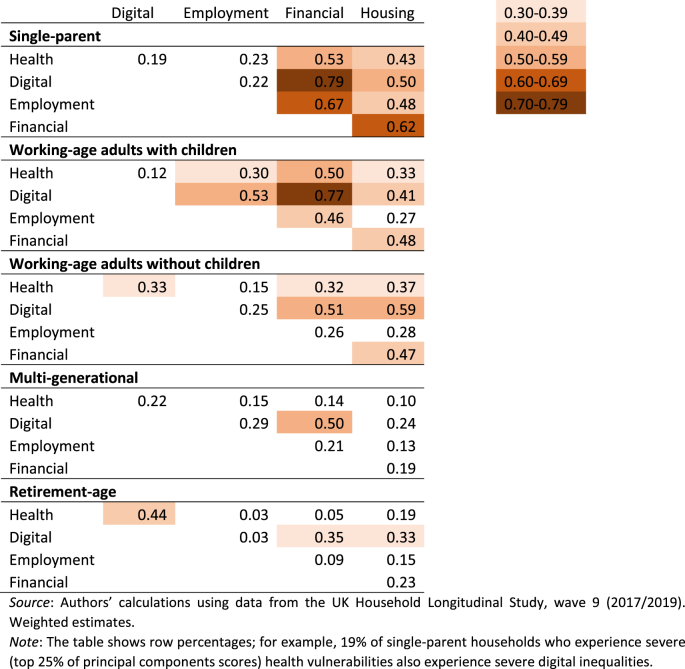
Proportion of different household types who experience intersecting severe vulnerabilities.

As a robustness check, we have replicated the PCA using individual-level data. We found the same five principal components identifying the same five dimensions of vulnerabilities and almost identical factor loadings. Then, we estimated weighted OLS regressions with clustering at the household-level to understand whether certain individual-level factors (i.e. age, sex, ethnicity, area type, marital status, and number of children) are associated with some types of vulnerabilities more than others in adjusted models (see Appendix Table A5). The findings are broadly in line what we know from the literature on socio-economic and health inequalities. The most novel aspects are the associations with COVID-19 health risks, which show that those from an ethnic minority background, who are unmarried, older, and those with low education are more likely to have those risks (controlled for other factors).

## Discussion

Our analysis highlights four key findings. First, while COVID-19 health risks are concentrated in retirement-age households, a substantial proportion (up to 25%) of working-age households also face these health risks. At the individual level, these health risks are also higher among those of ethnic minority background, who have low education, are older, and are unmarried.

Second, we show that all types of households are exposed to multiple, intersecting vulnerabilities. Financial and housing precarity is most prevalent among single-parent households, working-age adult households with children primarily face employment and financial insecurities, whereas their childless counterparts are most susceptible to housing deprivation and equally vulnerable on all other dimensions. Multi-generational households are likely to experience health and employment vulnerabilities, whereas retirement-age households are characterised by the prevalence of digital and health vulnerabilities. This emphasises that in multi-generational and retirement-age households, health risks co-exist with socio-economic vulnerabilities. This could mean that poor health, or the need to shield, could exacerbate existing financial precarities, or indeed that economic necessity could prevent households from self-isolating appropriately. Economic recession for households on the brink financially could worsen physical and social health conditions, thus making people even more vulnerable to the effects of COVID-19. We show that even in households where health-related risks are not as prevalent, different dimensions of socio-economic vulnerabilities co-exist. This highlights the importance of intersecting precarities, which may push vulnerable households towards poorer health outcomes.

Third, there is some variation in vulnerabilities across different areas of England and the constituent countries of the United Kingdom. This indicates that regional approaches may be considered when deciding on how to best mitigate the health and socio-economic consequences of the COVID-19 crisis. However, more spatial disaggregation is necessary to understand regional and neighbourhood-level vulnerabilities.

Fourth, in many households, different dimensions of vulnerabilities intersect. Among all household types and especially among working-age households, all dimensions of vulnerabilities intersect with financial and housing disadvantage. Among working-age households without children and among retirement-age households, severe health vulnerabilities intersect with severe digital disadvantage.

The results highlight the importance of household structure for the potential short- and long-term effects of the COVID-19 crisis. Future policy measures that aim to mitigate the socio-economic and health consequences of the COVID-19 crisis should consider the critical importance of household structure.

Household-level socio-economic and health vulnerabilities are likely to be context-specific, and thus the importance of households for moderating inequalities might vary across countries. For example, in Southern European countries, with a greater proportion of inter-generational co-residence and contact (Bayer & Kuhn, 2020; Dowd et al., 2020), household structure might matter in different ways, by making direct transmission to high risk individuals more likely. Similarly, the types of vulnerabilities that are relevant will vary between high- and low-income settings. In the United States, multidimensional poverty and race intersect at the individual, household, and neighbourhood scales. In low-income settings, household crowding and mixed generation households might pose serious barriers to the ability to shield elderly and vulnerable people, and these are likely to intersect with other dimensions of poverty (Dahab et al., 2020). Future studies should compare the importance of different dimensions of vulnerabilities by household type across European as well as low- and middle-income countries.

This study has some limitations. First, we likely underestimate the prevalence of health vulnerabilities as these measures are self-reported and not all members of the household have agreed to be interviewed. Additionally, our disease identification strategy is not specific enough to capture extremely vulnerable individuals who need to shield, because we do not know some of these precise conditions, nor do we know the severity of the conditions included in the analyses. In addition, further work is needed to explore patterns of multimorbidity, including mental health conditions, which are an additional vulnerability. Future work should repeat this analysis using linked health and administrative data that allow for identifying very high-risk individuals. Second, while this study was concerned with a description of intersectionality at the household level, future analysis of COVID-19 inequalities could take into account how different vulnerability dimensions are interrelated; for example, some portion of health vulnerability might be partially explained by socio-economic deprivation, which might suggest useful angles for social policy and prevention. Third, our analyses do not account for potential heterogeneity within households although additional individual-level analysis did show variations by factors such as ethnicity. Fourth, our analysis is based on data from 2017-19. However, we expect the relationships to be similar in 2020. Taken together, our study suggests that policy measures should take better account of intersecting household structure and dynamics for identifying vulnerabilities and advising citizens on how to deal with risks arising from the COVID-19 pandemic.

## Ethical statement

The analysis is based on anonymised secondary data from the UK Household Longitudinal Study. It does not require an ethical approval.

## Declaration of competing interest

We declare no conflicts of interest.

## Figures and Tables

**Table A1:** Share of different household types who experience the diseases in the COVID-19 health index

	Asthma		Heart failure		Heart disease		Angina		Myocardial Infarction		Stroke	

Proportion	SE	Proportion	SE	Proportion	SE	Proportion	SE	Proportion	SE	Proportion	SE

Single-parent	0.17	0.02	<0.01	0.00	<0.01	0.00	<0.01	0.00	0	.	<0.01	0.00
Working-age adults with children	0.22	0.01	<0.01	0.00	<0.01	0.00	<0.01	0.00	<0.01	0.00	<0.01	0.00
Working-age adults without children	0.18	0.01	<0.01	0.00	0.02	0.00	0.02	0.00	<0.01	0.00	<0.01	0.00
Multi-generational	0.22	0.01	0.02	0.00	0.07	0.00	0.07	0.00	<0.01	0.00	0.01	0.00
Retirement-age	0.13	0.01	0.03	0.00	0.08	0.00	0.08	0.00	0.01	0.00	<0.01	0.00
Total	0.17	0.00	0.01	0.00	0.04	0.00	0.04	0.00	<0.01	0.00	<0.01	0.00

	Emphysema		Chronic bronchitis		Liver condition		Cancer		Diabetes		Hypertension	
	Proportion	SE	Proportion	SE	Proportion	SE	Proportion	SE	Proportion	SE	Proportion	SE
Single-parent	<0.01	0.00	<0.01	0.00	0.03	0.01	<0.01	0.00	0.02	0.00	0.11	0.01
Working-age adults with children	<0.01	0.00	<0.01	0.00	0.03	0.00	0.02	0.00	0.03	0.00	0.11	0.01
Working-age adults without children	0.01	0.00	0.02	0.00	0.04	0.00	0.03	0.00	0.04	0.00	0.20	0.01
Multi-generational	0.03	0.01	0.05	0.01	0.05	0.01	0.09	0.01	0.12	0.01	0.44	0.02
Retirement-age	0.04	0.00	0.04	0.00	0.04	0.00	0.11	0.01	0.11	0.01	0.46	0.01
Total	0.02	0.00	0.02	0.00	0.03	0.00	0.05	0.00	0.06	0.00	0.27	0.00

*Source*: Authors' calculations using data from the UK Household Longitudinal Study, wave 9 (2017/2019). Weighted estimates.

**Table A2:** Percentage of households by household type where more than one person is vulnerable on individual-level indicators

	Unemployed	Part-time employed	Temporary contract	Self-employed	Long-term sick	COVID-19 health-risk
Single-parent	0.91%	3.03%	0.51%	0.20%	0.51%	1.62%
Working-age adults with children	0.84%	3.91%	0.93%	1.74%	0.15%	1.74%
Working-age adults without children	0.71%	2.82%	0.98%	1.43%	0.53%	2.47%
Multi-generational	0.60%	2.66%	0.79%	2.00%	0.12%	9.73%
Retirement-age	0.00%	0.39%	0.08%	0.29%	0.00%	6.10%

Total	0.56%	2.43%	0.70%	1.19%	0.28%	3.82%

*Source*: Authors' calculations using data from the UK Household Longitudinal Study, wave 9 (2017/2019). Weighted estimates.

**Table A3:** Share of different household types who experience the analysed vulnerabilities

	Living in a flat		Private renting		Overcrowding		No internet		No access to PC	

Proportion	SE	Proportion	SE	Proportion	SE	Proportion	SE	Proportion	SE

Single-parent	0.15	0.02	0.28	0.02	0.06	0.01	0.05	0.01	0.06	0.01
Working-age adults with children	0.08	0.01	0.15	0.01	0.16	0.01	0.01	0.00	0.02	0.00
Working-age adults without children	0.20	0.01	0.18	0.01	0.01	0.00	0.07	0.00	0.08	0.00
Multi-generational	0.05	0.01	0.06	0.01	0.02	0.00	0.06	0.01	0.07	0.01
Retirement-age	0.14	0.01	0.05	0.00	0.00	.	0.32	0.01	0.32	0.01

	Unemployed		Self-employed		Part-time employed		Temporary contract		Payment arrears	
	Proportion	SE	Proportion	SE	Proportion	SE	Proportion	SE	Proportion	SE
Single-parent	0.11	0.02	0.08	0.01	0.34	0.02	0.07	0.01	0.32	0.02
Working-age adults with children	0.07	0.01	0.19	0.01	0.42	0.01	0.12	0.01	0.11	0.01
Working-age adults without children	0.07	0.00	0.14	0.00	0.24	0.01	0.12	0.00	0.09	0.00
Multi-generational	0.06	0.01	0.15	0.01	0.28	0.01	0.11	0.01	0.03	0.01
Retirement-age	0.00	0.00	0.03	0.00	0.07	0.00	0.03	0.00	0.02	0.00

	Low income		COVID-19 health risk		Long-term sick					
	Proportion	SE	Proportion	SE	Proportion	SE				
Single-parent	0.30	0.02	0.16	0.02	0.07	0.01				
Working-age adults with children	0.12	0.01	0.17	0.01	0.03	0.00				
Working-age adults without children	0.15	0.01	0.26	0.01	0.11	0.01				
Multi-generational	0.10	0.01	0.49	0.02	0.10	0.01				
Retirement-age	0.16	0.01	0.49	0.01	0.01	0.00				

*Source*: Authors' calculations using data from the UK Household Longitudinal Study, wave 9 (2017/2019). Weighted estimates.

**Table A4:** Share of households across different areas who experience the analysed vulnerabilities

	Living in a flat		Private renting		Overcrowding		No internet		No access to PC	

Proportion	SE	Proportion	SE	Proportion	SE	Proportion	SE	Proportion	SE

North of England	0.09	0.00	0.13	0.01	0.03	0.00	0.15	0.01	0.15	0.01
South of England	0.13	0.01	0.14	0.01	0.03	0.00	0.11	0.01	0.11	0.01
London	0.39	0.02	0.20	0.01	0.08	0.01	0.11	0.01	0.11	0.01
Wales	0.09	0.01	0.12	0.01	0.02	0.01	0.15	0.01	0.16	0.01
Scotland	0.28	0.01	0.10	0.01	0.04	0.01	0.15	0.01	0.16	0.01
Northern Ireland	0.05	0.01	0.16	0.02	0.04	0.01	0.21	0.01	0.26	0.02

	Unemployed		Self-employed		Part-time employed		Temporary contract		Payment arrears	
	Proportion	SE	Proportion	SE	Proportion	SE	Proportion	SE	Proportion	SE
North of England	0.06	0.00	0.10	0.00	0.22	0.01	0.07	0.00	0.08	0.00
South of England	0.04	0.00	0.14	0.01	0.24	0.01	0.09	0.00	0.07	0.00
London	0.06	0.01	0.15	0.01	0.23	0.01	0.14	0.01	0.12	0.01
Wales	0.05	0.01	0.09	0.01	0.20	0.01	0.10	0.01	0.08	0.01
Scotland	0.05	0.01	0.10	0.01	0.21	0.01	0.08	0.01	0.07	0.01
Northern Ireland	0.08	0.01	0.09	0.01	0.21	0.01	0.07	0.01	0.11	0.01

	Low income		COVID-19 health risk		Long-term sick					
	Proportion	SE	Proportion	SE	Proportion	SE				
North of England	0.17	0.01	0.34	0.01	0.06	0.00				
South of England	0.13	0.01	0.33	0.01	0.05	0.00				
London	0.12	0.01	0.28	0.01	0.05	0.01				
Wales	0.17	0.01	0.33	0.02	0.07	0.01				
Scotland	0.17	0.01	0.30	0.01	0.08	0.01				
Northern Ireland	0.22	0.02	0.27	0.01	0.11	0.01				

*Source*: Authors' calculations using data from the UK Household Longitudinal Study, wave 9 (2017/2019). Weighted estimates.

**Table A5:** Results of individual-level OLS regressions on the predictors of the five dimensions (digital, financial, employment, health, housing) of vulnerabilities

	M1: Digital	M2: Financial	M3: Employment	M4: Health	M5: Housing
**Sex**					
Male (ref)					
Female	−0.023	−0.022	0.125∗∗∗	−0.025	−0.005
**Ethnicity**					
British (ref)					
Ethnic minority	0.021	0.387∗∗∗	0.063	0.066∗	0.330∗∗∗
**Area**					
North of England	0.046	−0.069	−0.137∗∗∗	0.024	−0.569∗∗∗
South of England	−0.096	−0.150∗∗	−0.030	0.000	−0.450∗∗∗
London (ref)					
Wales	0.027	−0.043	−0.054	0.020	−0.553∗∗∗
Scotland	0.108	−0.032	−0.088	0.088∗	−0.250∗∗∗
Northern Ireland	0.252∗∗∗	−0.042	−0.145∗∗	−0.005	−0.596∗∗∗
**Level of education**					
Low	0.335∗∗∗	0.290∗∗∗	−0.100∗∗∗	0.271∗∗∗	0.129∗∗∗
Medium (ref)					
High	−0.117∗∗∗	−0.159∗∗∗	0.018	−0.112∗∗∗	−0.003
**Marital status**					
Married/civil partnership (ref)					
Cohabiting	0.202∗∗∗	0.270∗∗∗	0.043	0.117∗∗∗	0.459∗∗∗
Widowed	1.423∗∗∗	0.190∗∗∗	−0.115∗∗∗	0.343∗∗∗	0.227∗∗∗
Separated/divorced	0.679∗∗∗	0.364∗∗∗	−0.038	0.473∗∗∗	0.479∗∗∗
Never married	0.693∗∗∗	0.447∗∗∗	−0.005	0.416∗∗∗	0.343∗∗∗
**Age**					
16–19	−0.610∗∗∗	0.035	0.329∗∗∗	−0.533∗∗∗	−0.598∗∗∗
20–29	−0.262∗∗∗	0.159∗∗	−0.015	−0.279∗∗∗	−0.150∗∗
30-39 (ref)					
40–49	0.114∗∗∗	−0.025	0.038	0.183∗∗∗	−0.340∗∗∗
50–59	0.222∗∗∗	0.088∗	0.164∗∗∗	0.324∗∗∗	−0.511∗∗∗
60–69	0.411∗∗∗	−0.002	0.060	0.318∗∗∗	−0.583∗∗∗
>70+	1.173∗∗∗	−0.106∗	−0.325∗∗∗	0.254∗∗∗	−0.685∗∗∗
**Number of children**					
None (ref)					
1 child	−0.053	0.283∗∗∗	0.130∗∗∗	−0.054	0.024
2 children	−0.021	0.541∗∗∗	0.202∗∗∗	−0.086∗∗	−0.061
3+ children	−0.100	1.608∗∗∗	0.141∗∗	−0.003	0.230∗∗∗
**Constant**	−0.610∗∗∗	−0.407∗∗∗	−0.007	−0.325∗∗∗	0.606∗∗∗
R-squared	0.253	0.169	0.045	0.084	0.134
N	27,625	27,625	27,625	27,625	27,625

*Source*: Authors' calculations using data from the UK Household Longitudinal Study, wave 9 (2017/2019). Weighted estimates. Standard errors have been adjusted to household-level clustering of individuals.

*Note*: ∗p ​< ​0.05; ∗∗p ​< ​0.01; ∗∗∗p ​< ​0.001.

## Footnotes

1 Of these 19,425 households, 83% has completed interviews with all eligible household members or provided proxy answers for those who did not provide a full interview. Additional analyses (not shown but available upon request) showed that the findings are very similar if we only use information from these households.

2 These conditions are asthma, arthritis, congestive heart failure, coronary heart disease, angina, heart attack or myocardial infarction, stroke, emphysema, hyperthyroidism, hypothyroidism, chronic bronchitis, liver condition, diabetes, cancer or malignancy, epilepsy, hypertension, clinical depression, and in some waves also multiple sclerosis, HIV, and other long standing/chronic conditions.

3 https://www.nhs.uk/conditions/coronavirus-covid-19/people-at-higher-risk-from-coronavirus/whos-at-higher-risk-from-coronavirus/.

4 As state pension age varies by respondents' birth year, UKHLS uses information on respondents' age at the time of interview to determine whether they were of state pension age following state pension rules applicable since 6 April 2016.

5 Varimax rotation leads to very similar factor loadings and identical principal components.
